# A Medical Hand-Book for the Use of Practitioners and Students

**Published:** 1894-03

**Authors:** 


*A Medical Hand-book for the Use of Practitioners and Students.* By R. S. Aitchison, M.B. Pp. xii., 347. London: Charles Griffin & Company, Limited. 1893.

While we have a strong belief in the utility of abstracts made by the reader himself in order to aid his memory, classify his facts, and assist revision, we do not think that abstracts made by other people are of much real service, and therefore we do not greatly favour the multiplication of small "hand-books" and abstract-books. The use of such "royal roads to learning" generally ends in giving a mere smattering of a subject, without any real knowledge of it. So far, however, as the present work is concerned, it seems to us to be well done, and free from most of the faults of these books. We have found the information given to be accurate, clearly put in a short compass, and the most important facts are given due prominence. Students beginning their duties as clinical clerks would find it helpful if used with, and not as a substitute for, the standard text-books.

